# The Lessons Taught by Suffering

**Published:** 1889-08-24

**Authors:** 


### Words of Consolation.

### THE LESSONS TAUGHT BY SUFFERING.

*(For Reading to the Sick.)*

Pain and suffering both of body and mind are in some mysterious way connected with sin, for no sooner had man fallen from the perfection in which God had made him than crime quickly followed, with all its attendant horrors of fear, misery, and shame. But though we have numerous examples even in the present day of swift vengeance falling on those who defy the majesty of God, yet we are not to suppose that all misery and suffering are direct punishments for sin. On the contrary, our Lord distinctly says it is not so. In answer to His disciples who brought before His notice the cases of the "Galileans whose blood Pilate mingled with their sacrifices," and the eighteen on whom the Tower of Siloam fell, he says, "Think ye they were sinners above all them that dwelt at Jerusalem? I tell you nay, but except ye repent ye shall likewise perish." We must use these "visitations of God" for our own warning, not for the condemnation of others.

Misery and suffering are great factors in the purification of the world. Many who are in the thick of the bustle of this world, who are making gods of themselves or of their wealth or of their brains; or, absorbed in their work, have no time to think of their souls or of their Saviour who bought them, these busy ones suddenly realise the truth of the words of the Psalmist—"Thou hidest Thy face, they are troubled; Thou takest away Thy breath, they die and return to their dust." God leads them into the wilderness and communes with them alone. In the quiet and rest of the sick-room, if they will but submit themselves to His will, they learn more and greater truths in a few weeks than years of health and prosperity would teach them. It has been well said, "We are in the hands of a higher Physician than this world knows; One who cannot mistreat our case or prescribe wrongly for us. The great cure to be wrought in us is the cure of self-will, that we may learn self-resignation; and all God's various dealings with us have this one end in view." Happy, thrice happy are they who use this time of rest for recollection and prayer, and, rising from illness to renewed life with the earnest intention of amendment, can in after years say "Before I was afflicted I went astray; but now have I kept Thy word."

Our Heavenly Father has special teaching for His faithful children besides those he gives through His word, His Church, His Sacraments, and the joys and sorrows of every day's experience, and they who frequent or watch by the bed of sickness and they who suffer, can see how wise and merciful are the various chastenings which He uses in fitting souls for eternal bliss. Christ, the true Vine, says of each living branch "He purgeth it that it may bring forth more fruit." Yes, in close communion with the "Captain of our salvation," who Himself "was made perfect through suffering," and in contemplating His sorrows we become like unto Him—"love, joy, peace, long suffering, gentleness, goodness, faith, meekness, temperance," all the fruits of the Spirit burst forth, and the softened heart declares "Thy rod and Thy staff they comfort me."

